# Risk Factors for Equine Gastric Ulcer Syndrome Incidence in Adult Icelandic Riding Horses

**DOI:** 10.3390/ani13223512

**Published:** 2023-11-14

**Authors:** Nanna Luthersson, Úndína Ýr Þorgrímsdóttir, Patricia A. Harris, Tim Parkin, Charlotte Hopster-Iversen, Euan D. Bennet

**Affiliations:** 1 Hestedoktoren, Hojgaard Sjaelland ApS, Hvalsovej 298, DK-4360 Eskilstrup, Denmark; nanna@hestedoktoren.dk; 2 Dyrlæge ehf, Vidarás 85, 110 Reykjavík, Iceland; 3 Equine Studies Group, Waltham Petcare Science Institute, Melton Mowbray LE14 4RT, UK; 4 Bristol Veterinary School, University of Bristol, Langford, Bristol BS40 5DU, UK; 5 Department of Veterinary Clinical Sciences, University of Copenhagen, DK-2630 Taastrup, Denmark; charlotte.hopster-iversen@sund.ku.dk; 6 School of Biodiversity, One Health, and Veterinary Medicine, University of Glasgow, Glasgow G61 1QH, UK

**Keywords:** EGUS, nutrition, forage, pasture

## Abstract

**Simple Summary:**

Previously, it was shown that Icelandic horses had a relatively high prevalence of gastric ulcers, in both regions of the stomach, which was found by performing gastroscopy on horses within two weeks of coming into training for the first time from the pasture. There was significant improvement in those in the squamous (non-glandular) region after eight weeks, especially for those being fed more frequent forage meals. This original study was undertaken in mainly young horses being trained for the first time. The current study evaluated the risk factors for Icelandic riding horses at various ages and stages of their training. This study found a low prevalence of gastroscopically significant squamous ulcers (grade ≥ 2/4: ~27%) in Icelandic horses being kept in training establishments and fed low starch and sugar diets. It was found that body condition, age and workload were not significantly associated with either squamous or glandular ulcer score but the region of Iceland wherein the horses were being kept did have an influence, as did their sex. Those animals showing clinical signs often associated with gastric ulcers were at increased risk of having gastroscopically significant glandular or gastroscopically severe squamous ulcers. It also highlighted the relatively high prevalence of ulcers in the glandular region (~46%) but did not identify any risk factors for such ulcers that could easily be modified.

**Abstract:**

A high prevalence of both squamous (ESGD) and glandular (EGGD) ulcers was previously found in, mainly young, Icelandic horses coming into training for the first time. This study evaluated risk factors for gastric ulcers in Icelandic riding horses at various ages and stages of training. The horses (n = 211) were gastroscoped from 21 equine establishments across Iceland. A variety of morphometric, clinical, behavioural and management factors were evaluated as potential risk factors for gastroscopically significant (grade ≥ 2/4: found in 27% of horses) or gastroscopically severe (grade 3 or 4/4: found in ~10% of horses) ESGD or gastroscopically significant EGGD (grade ≥ 1/2: found in 46.4%). Body condition score (BCS), cresty neck score (CNS), stable/turnout behaviour, exercise intensity/frequency and age were not significantly associated with ESGD or EGGD ulcer score. However, having come off the pasture into training for 4 weeks or less was a significant risk factor for gastroscopically significant and severe ESGD compared to 5 weeks or more. For both EGGD and ESGD, “region” was important. Gastroscopically significant EGGD and gastroscopically severe ESGD were more prevalent in those showing clinical signs often associated with ulcers. Geldings were more likely to have gastroscopically significant ESGD than both mares and stallions and more EGGD than stallions. Being stabled, but spending >2 h/day out in the paddock, compared with <2 h paddock time or full-time turnout, was protective for gastroscopically significant ESGD as was being fed complementary feed (all fed <1 g non-structural carbohydrate (NSC)/kg/BW/meal). Being at a training establishment for >4 weeks was protective for gastroscopically significant and gastroscopically severe ESGD but not EGGD. This study confirms the relatively low prevalence of ESGD in Icelandic horses being kept in training establishments and fed low NSC diets but highlights the high prevalence of EGGD.

## 1. Introduction

Equine gastric ulcer syndrome or EGUS [1,2,3,4] is the overriding term for the erosive and ulcerative pathology of the equine stomach, terminal oesophagus and proximal duodenum, which has the potential for a negative influence on welfare and performance [5,6,7,8]. The pathophysiology, risk factors and response to treatment primarily depend on the location of the ulcers. Currently, the term “Equine Squamous Gastric Disease (ESGD)” is used to describe ulcers present in the squamous mucosa of the stomach and “Equine Glandular Gastric Disease (EGGD)” is used for those found in the glandular part (i.e., cardia, glandular fundus, antrum, and pylorus). Depending on the methodology, the scoring system and the grades included, EGUS can be present in around 90% of actively training and exercising animals [2,3,4,9,10], and in up to 80% of pleasure riding horses [11]. A number of risk factors for ESGD have been identified, including exercise intensity, but in particular certain nutritional factors such as low fibre intake, long gaps between forage provision and the feeding of starch-rich complementary feeds [1,4,9,12]. Appropriate dietary changes, especially reducing the non-structural carbohydrate intake to <1 g/kg BW/meal, have been shown to be a beneficial management strategy for ESGD in practice [13]. There is less evidence, however, for the role of nutrition and management in EGGD where exercise intensity and in particular exercise frequency are considered to be more relevant [9,10,12,14,15].

Horses being housed and managed more intensively have, therefore, been considered to be more at risk of EGUS [9]. However, a recent study investigating, for the first time, the incidence of gastric ulceration in Icelandic horses in Iceland showed a comparatively high incidence of both ESGD (72% with an endoscopic grade of 2 or more/4) and EGGD (47% with a gastroscopic grade of 1 or 2/2) in animals coming from pasture [16]. There was a beneficial effect of being kept at a training establishment on the ESGD grade. This was associated, in particular, with being provided with more than three meals of forage/day. The authors suggested that, as “farm” represented around 35% of the variance in ESGD reduction, local management was likely to be a significant contributor to the likelihood of the ESGD score reducing. In this previous study [16], none of the management factors evaluated (which included the number of riders/week and the number of people feeding the horses) had an effect on the incidence of EGGD despite a relatively high proportion of the horses having glandular ulcers upon arrival (47%). The multi-variable analysis also failed to identify any managemental risk factors responsible for either EGGD reduction or increase over the 8 weeks of being at the training establishment with very light training.

The previously mentioned study [16] only evaluated naïve horses coming from pasture into light training for the first time for 8 weeks, during which time they were fed very little complementary feed. The aim of the current study, therefore, was to assess the prevalence and risk factors for EGUS in Icelandic horses at various stages of training. The aim was to include those that had been in training for a longer period of time and/or had undertaken repeated bouts of training and were potentially being fed higher intakes of complementary feed and exercised more intensively. The current study, therefore, looked at the prevalence of EGUS in a variety of adult Icelandic riding horses in order to further address these questions.

## 2. Materials and Methods

### 2.1. Animals

In this prospective study, 211 adult horses (age range 3–20 years) were recruited in 4 different regions of Iceland (North, West, South and around the capital Reykjavík) in the period from 25 November 2021 until 10 January 2022. None of these horses were included in the initial study [16] and all were described as riding horses by their owners/trainers. All horses had been out extensively grazing before arriving at the training establishment where they were gastroscoped. Sixty-three had been at the training establishment for 4 weeks or less prior to gastroscopy, whereas 148 had been stabled and trained for 5 weeks or more before the gastroscopy. 

### 2.2. Data Collection

Data including signalment, body condition score (BCS: out of 9: [17]), weight (with a horse-specific weight tape) and cresty neck score (CNS: out of 5; [18]) were recorded at the time of gastroscopy. Details regarding housing (type of box, No. of horses in stable and box), type of outdoor area used during the day, number of horses in outdoor area, as well as feeding (details of complementary feeds and forage provision as well as amount and times per day) and the number of riders/week and the number of people feeding the horses, were obtained by interviewing the trainer/owner (without their knowledge of the gastroscopy results). Estimated starch and sugar intakes were based on the details provided on the feed labels and the manufacturer’s information.

Details regarding training (intensity, duration, and times per week) were recorded. In addition, a further short questionnaire on the individual horse’s behaviour, regarding their level of reactivity (on a scale of 1–5) when being in a stable, out at pasture, handled and fed, was completed through interviewing the owners/trainers. Finally, the trainers were questioned regarding the presence or absence of various clinical signs (see Appendix A); some of which have been postulated to be associated with the presence of gastric ulcers in adult horses, including for ESGD: inappetence, poor body condition or weight loss, changes in behaviour, acute or recurrent colic, bruxism and stereotypic behaviour (crib biting, stall weaving) and poor performance (NL personal experience, [2,3,4,8,15]).

### 2.3. Gastroscopy

Horses were fasted for 15–18 h prior to gastroscopy but had free access to water. Light sedation was used (detomidine 8–10 microgram/kg and butorphanol 10–12 microgram/kg). A complete evaluation of the non-glandular region of the stomach was undertaken and a record of any lesions was made using the EGUS council severity scoring system (0–4/4; [2]). ESGD scores were determined on a scale from 0 to 4, with 0 being “no ulcers”, grades 2 or more being considered to be “gastroscopically significant” and a score of 3 or 4 being considered “gastroscopically severe”. EGGD scores were determined on a scale of 0–2 [19], where a score of 0 means that no ulcer is present or only hyperaemia, a score of 1 represented the presence of mild-to-moderate lesions (s) with evidence of loss of mucosal integrity and a score of 2 showing the presence of severe lesion (s) with evidence of loss of mucosal integrity. The presence of any EGGD ulcer was interpreted as being “gastroscopically significant” [19], and a score of 2 was interpreted as “gastroscopically severe” ulceration.

Owners were informed about the possible consequences of horses having gastroscopically significant or severe ulcers. The option of medical treatment for any ulcers found was discussed in detail with the owners as well as possible changes in feeding and management. All owners/trainers were instructed to contact their own veterinarian if a horse showed any clinical signs such as colic, lack of appetite and weight loss, regarding possible treatment.

### 2.4. Statistical Evaluations

Analysis was carried out in bespoke code written in R version 4.2.1 (R Foundation for Statistical Computing, www.r-project.org; accessed on 1 February 2023). The Tidyverse family of packages was used during initial data cleaning and sorting, and for figure and table production (https://joss.theoj.org/papers/10.21105/joss.01686; accessed on 1 February 2023).

Twenty-five variables were selected for analysis from the recorded data. The first stage of model-building was to construct a univariable logistic regression model for each variable. A threshold *p*-value of 0.2 was used to determine which variables were carried forwards into the multivariable analysis. The multivariable logistic regression model was constructed in a manual stepwise bidirectional process with the Akaike Information Criterion used to identify the best-fitting model at each step. The threshold *p*-value for inclusion in the final model was 0.05. Odds ratios and 95% confidence intervals were calculated for the interpretation of effect sizes. Variables rejected at the univariable and multivariable stages were assessed as potential confounders in the final model. The Hosmer–Lemeshow goodness-of-fit test was used to assess the overall fit of the final model. Farm name was included as a random effect in a mixed-effects model for comparison with the final single-level model. 

## 3. Results

All the horses were considered healthy with no previous diagnosis or treatment of gastric ulceration according to the owners/trainers. None of the horses had been treated with non-steroidal anti-inflammatory agents in the previous 3 months according to the owners/trainers.

### 3.1. Age, Body Condition (BCS) and Cresty Neck (CNS) Scores

According to their individual microchip information, 9% (n = 19) of the horses in the study were aged between 3 and 4 years; 47% (n = 100) were between 4 and 6 years old; 24% (n = 50) were between 7 and 9 years old; 13% (n = 27) were aged 10–14 years and 7% (n = 15) were between 15 and 20 years old.

A total of 32% of the horses (n = 68) had a BCS of 5/9, with 44% (n = 92) scoring 6/9, 22% (n = 47) scoring 7 and less than 2% (n = 4) scoring a BCS of 8/9. None of the horses had a BCS of less than 5/9.

For the cresty neck score, 70% (n = 147) of the horses scored a 2 and 29% (n = 62) scored a 3. One individual horse scored a CNS 1, and one other individual horse scored a CNS 4.

Neither BCS, CNS nor age were found to be significantly associated with any outcomes investigated during the present study.

### 3.2. Clinical Signs

Despite the horses being clinically healthy according to the owners/trainers, upon more in-depth questioning, 46/211 horses had non-specific clinical signs (Table 1) that have been associated with gastric ulcers [2,3,8,15,20,21]. Most of the horses showing such signs only showed one or two of the signs (20 in each category). Six horses showed three signs (ESGD scores of 2, 3, 0, 1, 0, 4 and EGGD scores of 2, 0, 2, 0, 1, 1) and only one horse showed four of the signs (ESGD score of 2 and EGGD score of 1).

### 3.3. Diet

Slightly above 70% of the horses were fed forage only (details missing for one horse) with 4.7% being fed <1 kg/100 kg BW/day and 32.2% being fed between 1 and 1.5 kg/100 kg BW/day. This meant that the majority of the horses were being fed at or more than 1.5 kg/100 kg BW per day (i.e., 1.5–2 kg/100 kg BW/day (35%); 2–2.5 kg/100 kg BW/day (17.5%); 2.5–3 kg/100 kg BW/day (2.4%)) and >3 kg/100 kg BW/day (7.1%)). The forage was being fed from the ground and no hay nets or forage extenders were used on any of the farms. 

Just under a third of the horses (29.4%) were fed some type of complementary feed in addition to the forage. Such complementary feeds were only provided once a day and for 80.6% of the horses the estimated sugar and starch intake was <0.5 g/kg BW/meal and the remainder were fed between 0.5 and 1 g/kg BW/meal. No horse was estimated to be fed more than 1 g/kg BW sugar+ starch/meal. 

### 3.4. Exercise

Although intensity and frequency were recorded separately, the intensity was kept constant for the number of days that the animals were exercised by each trainer per week at each stage of training. The number of days exercised/week at each intensity level was also constant between the trainers, and therefore a combined definition of workload (combination of intensity and frequency) was also applied. At the time of scoping, 33/211 horses were in little/no work (0–1 days per week), 59/211 were in light work (2–3 days per week), 117/211 were in moderate work (4–5 days per week) and 2/211 were in hard work (6–7 days per week).

### 3.5. ESGD

Just over a quarter of the horses had gastroscopically significant (≥2/4) ulcers (57/211: 27%), with 8.5% having gastroscopically severe ulcers (≥3/4) (Table 2). The prevalence in relation to the farm is shown in Table 3. 

Behaviour in the stable or during turnout was not significantly associated with ESGD in the final models, and neither were exercise frequency and intensity, alone or combined (i.e., an indication of workload). The majority of the horses were fed three or more forage meals a day (136/211) with the majority of the remainder being fed two meals per day (74/211). The number of forage meals was not a significant risk factor for ESGD.

**Figure 1:**
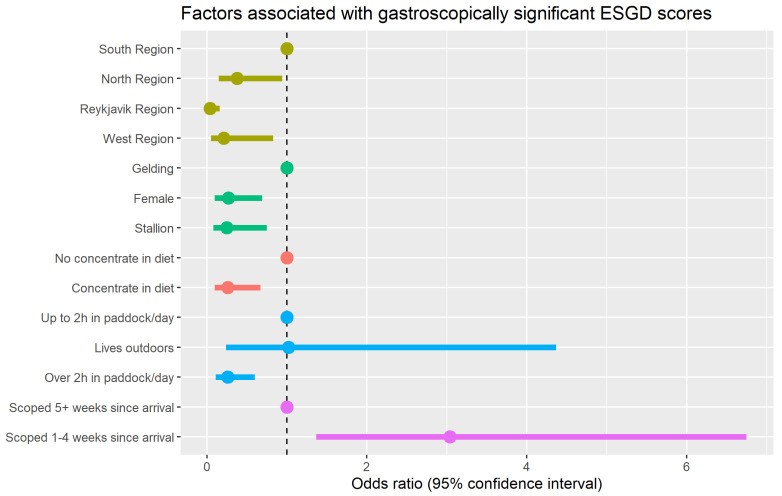
Dot-and-whisker plot showing the results of the multivariable model of factors associated with an ESGD score of 2 or higher/4. Odd ratios greater than one indicate increased risk and those that are less than one indicate reduced risk.

Five risk factors were identified as being associated with increased odds of gastroscopically significant ESGD (score of 2, 3 or 4/4). Table 4 and Figure 1 show the full results of the multivariable model for this outcome: (1) Horses in the south region were significantly more likely to have gastroscopically significant ESGD (≥2/4) than horses in any of the other three regions. (2) Geldings were significantly more likely to have gastroscopically significant ESGD (≥2/4) than either mares or intact males. (3) Horses that were fed a complementary feed as part of their ration were at reduced risk of having gastroscopically significant ESGD. (4) Horses that were stabled some of the time but spent more than 2 h per day in the paddock were at reduced odds of gastroscopically significant ESGD compared to those that spent less than 2 h per day in the paddock. (5) Horses that had arrived at the training establishment four weeks or less before scoping had a higher risk of having gastroscopically significant ESGD compared to those that had arrived five weeks or more before scoping.

**Figure 2:**
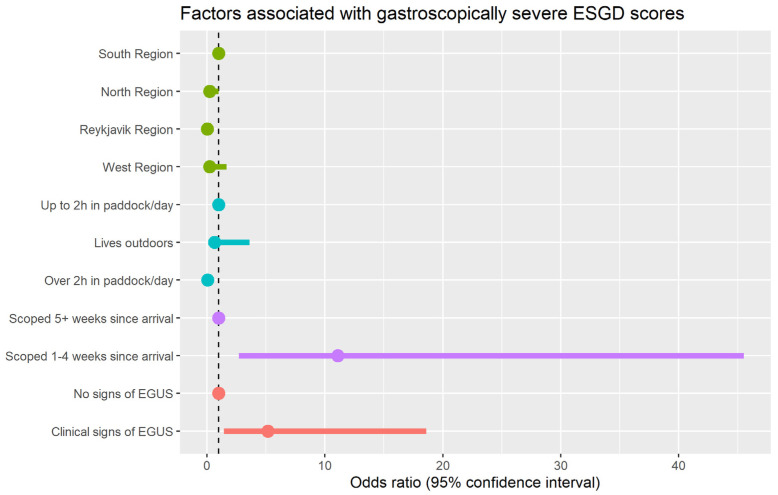
Dot-and-whisker plot showing the results of the multivariable model of factors associated with ESGD score of 3 or 4/4. Odd ratios greater than one indicate increased risk and those that are less than indicate one reduced risk.

Evaluating the risk factors for gastroscopically severe ESGD (score of 3 or 4/4), the following four risk factors were identified (Table 5) and illustrated in Figure 2: (1) Horses in the south region were at a greater risk than those in the north and Reykjavik regions. (2) Horses that were stabled for part of the day but spent less than 2 h per day in the paddock were at more risk than those who were also stabled part of the day but spent more than 2 h per day out in a paddock. (3) Horses that had arrived at the training establishment more recently before scoping were at significantly increased odds of severe ESGD. (4) Finally, horses exhibiting potential clinical signs of EGUS were at significantly increased odds of gastroscopically severe ESGD compared to those without clinical signs.

The farm or training establishment accounted for 56% of the variance with respect to the gastroscopically significant ESGD, and 36% of the variance for the gastroscopically severe ESGD model.

### 3.6. EGGD

**Figure 3:**
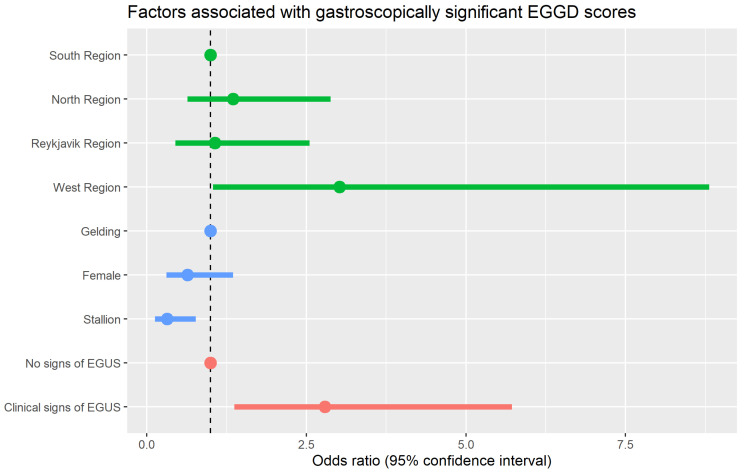
Dot-and-whisker plot showing the results of the multivariable model of factors associated with an EGGD score 1 or 2/2. Odd ratios greater than one indicate increased risk and that are those less than indicate one reduced risk.

Just under half the horses had gastroscopically significant EGGD, with 25 (11.8%) having gastroscopically severe (grade 2/2) EGGD (Table 1). Table 6 shows the results of the multivariable model for gastroscopically significant EGGD, which are illustrated in Figure 3. Three risk factors were shown to be associated with a gastroscopically significant EGGD score: (1) Horses in the west region were at significantly increased odds of significant EGGD compared to horses in the south region. (2) Stallions were at significantly reduced odds compared to geldings. (3) Horses exhibiting potential clinical signs of EGUS were at increased odds compared to horses with no clinical signs. No other factors, including exercise intensity, frequency and combined workload, were found to be associated in the final model.

Farm or training establishment accounted for 16% of the variance in the gastroscopically significant EGGD model.

## 4. Discussion

The present study showed a similar percentage of Icelandic horses with gastroscopically significant ESGD (27%) compared to a previous study [10] with naïve horses after 8 weeks of light training (25%); although there were three horses with grade 4 ulcers in the present study compared to none of the naïve animals in the previous study. This suggests that around 25% of Icelandic horses in training in Iceland, under such management conditions, may have gastroscopically significant ESGD ulcers. This is considerably lower than previous findings in other horses in training [2,4], where prevalences between 57 and 100% have been reported. This and the previous study were carried out in Iceland [16], which were the first times that gastroscopy had been undertaken in Iceland.

This lower prevalence may reflect the greater amount of forage fed (given that the majority were fed 1.5 kg or more of forage/100 kg BW per day), and the relatively low intake of starch per meal (none fed >1 g/kg BW/meal or per day), which may represent a reduced risk in the present study [8,9]. The relatively low incidence could also be a result of the lower intensity of training of Icelandic horses compared to the study population in other studies. In the current study, over 50% were in moderate work (4–5 days per week) and only two animals (2/211) were in hard work (6–7 days per week). Therefore, although workload was not a significant factor for ulcer incidence in the present study, further work in horses in hard and intensive work is required to further evaluate this for both ESGD and EGGD. This is important given that, whilst exercise intensity is important for ESGD, intensity as well as frequency is perhaps more relevant for EGGD [2,4]. It should be highlighted, however, that the incidence of gastroscopically significant EGGD was still relatively high in this study, as for the first study, with close to 50% of the horses having an EGGD score of 1 or 2/2. It is also important to note that horses showing clinical signs often associated with gastric ulcers (NL personal observation) were also more likely to have gastroscopically severe ESGD or gastroscopically significant EGGD. The three horses with grade 4/4 ESGD ulcers had all shown signs consistent with signs of pain (negative behaviour when ridden and reduced appetite) for some weeks. It is important to appreciate that this does not mean that horses showing these rather general signs of pain (e.g., negative behaviour when girthing up or riding, repeated minor colic signs, reduced appetite) will have ulcers, nor that animals without these signs will not have ulcers, nor that, if ulcers are present with signs of pain on riding [22,23], the ulcers are the primary cause of the pain. However, it does highlight the importance of a thorough clinical history and clinical examination. In the presence of potential clinical signs, a gastroscopy should be considered to confirm or exclude gastric ulcers as a potential contributor to these clinical signs. Gastroscopy plays an important role in any examination of horses showing signs of pain or reduced performance, and this study highlights the need to undertake gastroscopy to confirm both the presence of, and recovery from, ulcers. 

Interestingly, in this current study the amount/frequency of forage feeding did not have any effect on the risk of having ESGD. This may reflect not only that the majority were being fed sufficient forage, but that the majority of animals were also being fed their forage in at least three meals per day. Only 11/211 received less than 1 kg/100 kg BW/day of preserved forage and most of these horses were out on grass for some hours during the day and were in good body condition. It is also important to recognise that the low incidence of ulcers occurred despite the fact that 184/211 horses were fed their last evening meal of forage around 12–14 h before their early morning forage meal. This is not atypical of the way that horses are fed in general in Iceland. This perhaps seems contradictory to the general advice to keep the intervals between forage meals to a maximum of 4–6 h [4,9]. The work by Husted and colleagues [24] also showed that proximal gastric pH reduced during the early hours of the morning, whereas ventral pH remained fairly constant. The low incidence may also be related to the fact that the extended period without forage provision occurred during the night, when the majority of the horses were standing or lying quietly in their stables, resulting in limited “splashing” of any gastric liquid. Certainly, resting behaviour has been shown to peak at night between 9 pm and 4 am regardless of turnout or management conditions [25]. Previous observations in the field have shown that many stabled animals, provided with forage consistently or frequently throughout the day, that are not intensively exercised and are fed low or restricted starch and sugar meals, do not have any gastroscopically significant gastric ulceration despite also having long periods of time over the night without forage being available (PH/NL personal observations). It is possible that some may forage in their bedding depending on its type, but this is not the case for all. Whilst ad libitum forage may be the preferred option for forage provision [26], this is not always possible for practical and weight management reasons. Therefore, it is important to recognise that perhaps forage provision throughout the day may be more important under such circumstances. No gastroscopically significant (and very few in total) gastric ulcers were seen, for example, in prolonged forage-restricted ponies kept on rubbing matting when their time spent foraging was extended during the day [27]. Further work is required in this area, as it is very important practically. 

The feeding of complementary feed did have a protective effect in the present study which again may be seen as contrary to current recommendations. However, importantly, all horses that were being fed were being fed complementary feeds with a low NSC content. All were being fed lower estimated intakes of starch and sugar than is currently recommended, i.e., <1 g/kg BW/meal. It may also be that providing complementary feed was a proxy for more attention being paid to these animals. The implementation of management factors, not monitored in this study, therefore, could be of value in reducing the risk of ESGD. Interestingly, the feeds that were being fed did not contain specific acid buffers to help reduce gastric pH and were not low in starch and sugar, high fibre, high oil feeds that may be considered to be of the type and format to specifically help support gastric health [9]. They were instead based on processed grains combined with fibre sources and fed as muesli or a pellet. However, as mentioned before, they were only fed at low intake levels, which meant that they only provided very low NSC intakes.

Whilst several studies have shown an increase in ESGD with stabling and training [28,29,30], this has not always been the case [16,31]. Consistent with the reduction in ulcers seen in the initial study, following coming off pasture and coming to the training establishment [16], the time since arrival before being scoped in this current study also influenced the likelihood of having gastroscopically significant ulcers. The animals being scoped more than 5 weeks post-arrival were less likely to have gastroscopically significant and gastroscopically severe ESGD ulcers than those being examined 4 weeks or less after arrival. This also suggests that the relatively high percentage of ulcers in the naïve population coming into training is a reflection of the management/pasture, etc., when out in the herds, and an advantage of coming into a training establishment and being fed preserved forage (and possibly being housed for part of the day, as discussed below). Further work is needed on this aspect, especially as horses in Iceland are often out on pastures for long periods every year, during either summer or autumn, before coming into the stable and being ridden. 

Interestingly, being stabled for part of the day but kept outside for more than 2 h a day was apparently protective for gastroscopically significant and severe ESGD. This suggests that, potentially, some access to areas where the horses can move freely/potentially interact directly with other conspecifics and have access to forage may be beneficial [4]; whereas permanent maintenance outside may be detrimental. Being stabled for part of the day may also provide benefits, such as protection from adverse environmental conditions, individual feeding and access to forage without disturbance. Certainly, this suggests that further work looking at demeanour as well as conspecifics interactions and variation in vigilant behaviour may be beneficial when evaluating risk factors for gastric ulcers. In addition, these findings may hint at a possible detrimental factor in the pastures in Iceland, which should be evaluated further, especially as “region” was identified as an influencing factor. It is also important to note that other studies have also shown that pasture is not always protective [32,33,34]. More work is evidently needed to determine the reasons for this.

In this current study, the horses from the south region were more likely to have gastroscopically severe ESGD ulcers than those from the Reykjavik region. Those from the south were, however, less likely to have gastroscopically significant EGGD than those from the west. This was similar to the initial study which suggested that horses from the south were less likely to have a high EGGD score but were more likely to have a high ESGD score. However, in the first study, it was found that “region” was a confounder when the horses were scoped post-arrival. For example, 61% of horses scoped within 1–3 days of arrival were in the north region, while 88% of horses scoped within 4–6 days of arrival were in the south region. In the current study, “region” and “time of arrival” were both independently significant, and horses being scoped within 4 weeks of arrival for training were much more likely to have gastroscopically significant as well as gastroscopically severe ESGD. This work, therefore, supports the findings of the first study according to which bringing in Icelandic horses from pasture can be beneficial for their gastric (at least squamous) health. Looking at rainfall information from local weather stations for the different regions did suggest that, overall, the south regions (three stations: 364, 501 and 498 mm) had higher rainfall than the other regions for the 3 months previous to this study being undertaken. The north (three stations: 259, 234 and 257 mm) and west (one station: 254 mm) had similar and the lowest rainfall levels. The Reykjavik region received an intermediate amount (385 mm). Whether such environmental differences may impact pasture growth and species type needs to be confirmed. 

The farm accounted for over 50% of the variance with respect to the gastroscopically significant ESGD, and nearly 40% of the variance for the gastroscopically severe ESGD model, suggesting that, as per the initial study, “farm” seems to be a major influencing factor for ESGD. This suggests that local management is a significant factor above and beyond the factors that could be evaluated in this study. The management factors that were evaluated included the number of people feeding the horses and the number of riders per horse, which have previously been suggested to be potential risk factors [35]. However, for EGGD, “farm” only accounted for 16% of the variance, suggesting that local management is perhaps less important for EGGD––although, as mentioned above, “region” was a significant factor. Further work in larger numbers of farms may be required to evaluate the effect of region in combination with the farm. For example, depending on the level of ulceration upon arrival from the pasture, local management factors may influence how rapidly the ESGD ulcers heal.

Interestingly, sex was a significant factor in the prevalence of both gastroscopically significant ESGD and EGGD. Stallions and mares/fillies (i.e., females) were more likely to have lower ESGD ulcer scores compared to geldings in the initial study [16]. This suggests either a direct sex effect or the possibility that this is a proxy for some unidentified management factor(s), as geldings are potentially less valuable than females and stallions. Several studies have shown geldings at a higher risk for gastric ulcers than mares, although this is not consistent, as outlined in the study by Vokes and colleagues [4]. Interestingly, in contrast to the previous study in Iceland [16], sex did have an influence on EGGD score, although again geldings were more likely to have the higher scores. This does suggest that there may be some factors related either to sex, or potentially to management/feeding, that may contribute to EGUS at least in these Icelandic horses.

Whilst this study looked at the prevalence of, and risk factors for, gastric ulcers in general riding Icelandic horses under field conditions in Iceland, the number of horses in hard/intensive work was still very small. This makes it difficult to assess the relative importance of workload. Although over half the horses evaluated were in moderate or intensive work, none of them were being fed high intakes of starch and sugar. Therefore, the relative importance of NSC intake on ulcer prevalence could not be assessed. It would be interesting, therefore, to repeat the study at a different time of year when the horses had been in training for longer and potentially more would be in hard work.

## 5. Conclusions

Despite these limitations, this study confirms the findings of the initial study on naïve horses entering training for the first time, according to which being stabled and exercised does not automatically mean a high ulcer incidence. It also suggests that being extensively grazed at least during the autumn/winter increases the risk of gastroscopically significant and severe ESGD. It also confirms the relatively low prevalence of ESGD in Icelandic horses being kept in training establishments in Iceland and fed low NSC diets but highlights the high prevalence of EGGD. Importantly, as with other studies, no modifiable risk factors were identified for EGGD other than perhaps moving region. Whether sex is a direct risk factor for EGUS, or is associated with other indirect management factors, needs to be evaluated further.

## Figures and Tables

**Table 1:** Prevalence of clinical signs that may be associated with the presence of gastric ulcers.

Clinical Sign	Number (% of the 46 Horses Exhibiting Such Clinical Signs)
Negative behaviour when being groomed	24 (52.2%)
Girthiness	24 (52.2%)
Weight loss/picky eater	7 (15.2%)
Negative behaviour when ridden	13 (28.3%)
Crib biting	1 (2.2%)
Colic symptoms > 2 times in last 3 months	7 (15.2%)
Unwilling to go forward	4 (8.7%)
Horses exhibiting multiple clinical signs	
2 clinical signs	19 (41.3%)
3 clinical signs	6 (13.0%)
4 clinical signs	1 (2.2%)

**Table 2:** Descriptive statistics of the full cohort for both ESGD score and EGGD score.

ESGD Score	No. Horses (%)	EGGD Score	No. Horses (%)
0	110 (52.1%)	0	113 (53.6%)
1	44 (20.9%)	1	73 (34.6%)
2	39 (18.5%)	2	25 (11.8%)
3	15 (7.1%)		
4	3 (1.4%)		

**Table 3:** Descriptive statistics of horses at each farm in the study, including whether or not the horses had gastroscopically significant or gastroscopically severe ESGD scores. Different letters within the same column reflect a significant difference between regions.

			Gastroscopically Significant or Gastroscopically Severe ESGD	
Farm (Region)	No. Horses	Score (0 or 1/4)	Score 2/4 (Significant)	Score 3 or 4/4 (Severe)
A (North)	13	9 (69.2%)	3 (23.1%)	1 (7.7%)
B (North)	12	7 (58.3%)	2 (16.7%)	3 (25%)
C (North)	16	13 (81.2%)	3 (18.8%)	0 (0%)
D (North)	5	4 (80%)	1 (20%)	0 (0%)
E (North)	1	0 (0%)	1 (100%)	0 (0%)
F (North)	14	13 (92.9%)	1 (7.1%)	0 (0%)
G (North)	13	6 (46.2%)	5 (38.5%)	2 (15.4%)
H (Reykjavik)	9	8 (88.9%)	1 (11.1%)	0 (0%)
I (Reykjavik)	12	10 (83.3%)	2 (16.7%)	0 (0%)
J (Reykjavik)	2	2 (100%)	0 (0%)	0 (0%)
K (Reykjavik)	4	4 (100%)	0 (0%)	0 (0%)
L(Reykjavik)	5	5 (100%)	0 (0%)	0 (0%)
M (Reykjavik)	14	12 (85.7%)	1 (7.1%)	1 (7.1%)
N (Reykjavik)	1	1 (100%)	0 (0%)	0 (0%)
O (South)	8	5 (62.5%)	3 (37.5%)	0 (0%)
P (South)	22	21 (95.5%)	0 (0%)	1 (4.5%)
Q (South)	8	3 (37.5%)	5 (62.5%)	0 (0%)
R (South)	20	6 (30%)	8 (40%)	6 (30%)
S (South)	6	6 (100%)	0 (0%)	0 (0%)
T (South)	7	4 (57.1%)	1 (14.3%)	2 (28.6%)
U (West)	19	15 (78.9%)	2 (10.5%)	2 (10.5%)
Totals by Region				
North	74	52 (70.3%)	16 (21.8%) ^a^	6 (8.1%) ^A^
Reykjavik	47	42 (89.4%)	4 (8.5%) ^a^	1 (2.1%) ^A^
South	71	45 (63.4%)	17 (23.9%) ^b^	9 (12.7%) ^B^
West	19	15 (78.9%)	2 (10.5%) ^a^	2 (10.5%) ^AB^

**Table 4:** Results of a multivariable model showing factors associated with an ESGD score of 2 or higher/4. Odd ratios greater than one indicate increased risk and those that are less than one indicate reduced risk. * Refers to the reference factor within the risk category.

Risk Factor	Horses Scoped	Horses with ESGD Score of >1 (%)	Odds Ratio	95% Confidence Interval	*p*-Value
**Region**					
South *	71	26 (36.6%)	1	-	-
North	74	22 (30.0%)	0.38	0.15–0.94	0.04
Reykjavik	47	5 (10.6%)	0.04	0.01–0.16	<0.001
West	19	4 (21.1%)	0.21	0.05–0.83	0.03
**Sex**					
Gelding *	68	22 (32.4%)	1	-	-
Female	100	27 (27.0%)	0.27	0.1–0.69	0.007
Stallion	43	8 (18.6%)	0.25	0.08–0.75	0.01
**Complementary feed/concentrate as part of ration**					
No *	149	49 (32.9%)	1	-	-
Yes	62	8 (12.9%)	0.26	0.10–0.67	0.005
**Time spent in paddock per day–otherwise stabled**					
Up to 2 h * and otherwise stabled	120	33 (27.5%)	1	-	-
Over 2 h but otherwise stabled	79	17 (21.5%)	0.26	0.11–0.60	0.002
Always outdoors	12	7 (58.3%)	1.02	0.24–4.37	0.98
**Weeks since arrival at the training establishment**					
5 or more *	148	30 (20.3%)	1	-	-
0 to 4	63	27 (42.9%)	3.04	1.37–6.75	0.006

**Table 5:** Results of a multivariable model showing factors associated with an ESGD score of 3 or 4/4. Odd ratios greater than one indicate increased risk and those that are less than one indicate reduced risk. * Refers to the reference factor within the risk category.

Risk Factor	No. Horses Scoped	Horses with ESGD Score of 3 or 4 (%)	Odds Ratio	95% Confidence Interval	*p* Value
**Region**					
South *	71	9 (12.7%)	1	-	-
North	74	6 (8.1%)	0.22	0.05–0.98	0.047
Reykjavik	47	1 (2.1%)	0.03	0–0.32	0.004
West	19	2 (10.5%)	0.24	0.03–1.66	0.15
**Time spent in paddock per day**					
Up to 2 h *	120	13 (10.8%)	1	-	-
Over 2 h	12	3 (25.0%)	0.07	0.01–0.46	0.005
Always outdoors	79	2 (2.5%)	0.65	0.12–3.61	0.62
**Weeks since arrival at the training establishment**					
5 or more *	148	6 (4.1%)	1	-	-
0 to 4	63	12 (19.1%)	11.09	2.70–45.52	<0.001
**Signs of EGUS**					
No *	165	9 (5.5%)	1	-	-
Yes	46	9 (20.0%)	5.16	1.43–18.59	0.012

**Table 6:** Results of a multivariable model showing factors associated with EGGD score of 1 or 2/2. Odd ratios greater than one indicate increased risk and those that are less than one indicate reduced risk. * Refers to the reference factor within the risk category.

Risk Factor	No. Horses Scoped	Horses with EGGD Score > 0 (%)	Odds Ratio	95% Confidence Interval	*p* Value
**Region**					
South *	71	27 (38.0%)	1	-	-
North	74	36 (48.7%)	1.35	0.64–2.88	0.431
Reykjavik	47	23 (48.9%)	1.07	0.45–2.55	0.878
West	19	12 (63.2%)	3.02	1.04–8.81	0.042
**Sex**					
Gelding *	68	40 (58.8%)	1	-	-
Female	100	45 (45%)	0.64	0.31–1.35	0.246
Stallion	43	13 (30.2%)	0.32	0.13–0.77	0.011
**Signs of EGUS**					
No *	165	67 (40.6%)	1	-	-
Yes	46	31 (67.4%)	2.79	1.37–5.72	0.005

## Data Availability

Data are contained within the article.

## Appendix A. Questions Included with Respect to Clinical Signs

**Clinical Signs of EGUS**	**Yes or No**	**Comments**
Negative behaviour when being groomed		
Negative behaviour during saddling up and girthing		
Negative behaviour when ridden (bucking, tail-swishing, stopping)		
Reduced performance compared to owner expectation		
Unwilling to go forward when ridden		
Colic episodes (>2 within the last 3 months)		
Diarrhoea (within the last 3 months)		
Problems maintaining weight during the last 3 months		
Crib biting		
